# The Ganglioside GM3 Is Associated with Cisplatin-Induced Apoptosis in Human Colon Cancer Cells

**DOI:** 10.1371/journal.pone.0092786

**Published:** 2014-05-14

**Authors:** Tae-Wook Chung, Hee-Jung Choi, Seok-Jo Kim, Choong-Hwan Kwak, Kwon-Ho Song, Un-Ho Jin, Young-Chae Chang, Hyeun Wook Chang, Young-Choon Lee, Ki-Tae Ha, Cheorl-Ho Kim

**Affiliations:** 1 Molecular and Cellular Glycobiology Laboratory, Department of Biological Science, SungKyunKwan University, Suwon City, Kyunggi-Do, Republic of Korea; 2 Division of Applied Medicine, School of Korean Medicine, Pusan National University, Yangsan City, Gyeongsangnam-Do, Republic of Korea; 3 Research Institute of Biomedical Engineering and Department of Medicine, Catholic University of Daegu School of Medicine, Daegu, Republic of Korea; 4 Faculty of Pharmacy, Yeungnam University, Kyungsan, Republic of Korea; 5 Faculty of Medicinal Biotechnology, Dong-A University, Busan, Republic of Korea; Vanderbilt University School of Medicine, United States of America

## Abstract

Cisplatin (*cis*-diamminedichloroplatinum, CDDP) is a well-known chemotherapeutic agent for the treatment of several cancers. However, the precise mechanism underlying apoptosis of cancer cells induced by CDDP remains unclear. In this study, we show mechanistically that CDDP induces GM3-mediated apoptosis of HCT116 cells by inhibiting cell proliferation, and increasing DNA fragmentation and mitochondria-dependent apoptosis signals. CDDP induced apoptosis within cells through the generation of reactive oxygen species (ROS), regulated the ROS-mediated expression of Bax, Bcl-2, and p53, and induced the degradation of the poly (ADP-ribosyl) polymerase (PARP). We also checked expression levels of different gangliosides in HCT116 cells in the presence or absence of CDDP. Interestingly, among the gangliosides, CDDP augmented the expression of only GM3 synthase and its product GM3. Reduction of the GM3 synthase level through ectopic expression of GM3 small interfering RNA (siRNA) rescued HCT116 cells from CDDP-induced apoptosis. This was evidenced by inhibition of apoptotic signals by reducing ROS production through the regulation of 12-lipoxigenase activity. Furthermore, the apoptotic sensitivity to CDDP was remarkably increased in GM3 synthase-transfected HCT116 cells compared to that in controls. In addition, GM3 synthase-transfected cells treated with CDDP exhibited an increased accumulation of intracellular ROS. These results suggest the CDDP-induced oxidative apoptosis of HCT116 cells is mediated by GM3.

## Introduction

Cisplatin (*cis*-diamminedichloroplatinum, CDDP) is a commonly used chemotherapeutic agent for the treatment of several solid tumors. CDDP binds to DNA to generate DNA adducts [1]. Previous studies have showed that CDDP regulates the activity of certain ion channels, transport proteins and various plasma membrane enzymes [2], [3], and induces reactive oxygen species (ROS) during cancer chemotherapy [4]. Consequently, CDDP regulates DNA repair, transcription inhibition, cell cycle arrest and apoptosis [5]. It has been reported that the extracellular signal-regulated kinases (ERK), the c-Jun N-terminal kinases (JNKs)/stress-activated protein kinase (SAPK) and the p38 mitogen-activated protein kinase (MAPK) are activated in CDDP-induced apoptosis of cancer cells [6], [7]. Additionally, CDDP induces Fas/FasL-mediated apoptosis in human epithelial cells [8] and mitochondrial damage by regulating expression levels of the Bcl-2 family, which have either pro- or anti-apoptotic functions. By affecting CDDP cytotoxicity in cancer cells, p53-mediated transactivation of the pro-apoptotic Bax, decreased Bcl-2 expression and cleavage of Bid leads to a reduction in mitochondrial potential and increases caspase-mediated PARP degradation through the release of cytochrome c from mitochondria [9]–[12]. Several studies have reported that these apoptotic phenomena are due to the alteration of membrane composition [13]. Among the membrane components, it has been reported that expression levels of gangliosides are altered in drug-treated cancer cells [14].

Clusters of sialic acid-containing glycosphingolipids (GSLs) are ubiquitous in the membrane microdomain in all mammalian cells and are implicated in a broad range of biological functions, including cell-cell interactions and signal transduction [15]. Many studies have reported that specific gangliosides such as GM3, GD3, and GD1b induce apoptosis in various types of cells [16], [17]. GM3 treatment in immature proliferating glial and neuronal cells results in suppression of cell proliferation and the induction of apoptosis [18], [19]. Additionally, GM3 is involved in cell death through the accumulation of ROS and intracellular calcium ion influx into the neuronal cells [20], [21]. In murine bladder cancer cells, GM3 overexpression induces apoptosis and reduces malignant potential [22]. In the present study, we investigated the functional significance of ganglioside GM3 in CDDP-treated colorectal cancer cells.

## Materials and Methods

### Cell culture and Reagents

The human colon cancer cell line HCT116 was cultured in Dulbecco's modified Eagle's medium (DMEM; JBI, Daegu, Korea) supplemented with 10% fetal bovine serum (FBS), 100 units/mL penicillin and 100 µg/mL streptomycin at 37°C under 5% CO_2_. Cisplatin was purchased from Dong-A pharmaceutical CO., LTD (Seoul, Korea). *N*-acetyl-_L_-cysteine (NAC) was obtained from Molecular Probes™ (Invitogen, CA). Baicalein was from Sigma-Aldrich. Antibodies were obtained as follows: Anti-poly (ADP-ribosyl) polymerase (PARP), BCL-2, Bax, and p53 were obtained from Santa Cruz Biotechnology (Santa Cruz, CA). Anti-glyceraldehyde-3-phosphode-hydrogenase (GAPDH) was purchased from Chemicon (Temecula, LA). Anti-GM3 (M2590) was purchased from Biotest Laboratories (Japan).

### Reverse transcription-polymerase chain reaction (RT-PCR)

Total RNA from each cell was isolated using the Trizol reagent (Invitrogen). One microgram of RNA was subjected to reverse transcription with oligo dT primers using AccuPower RT-PreMix (Bioneer Co., Daejon, Korea), according to the manufacturer's protocol. The cDNA was amplified by PCR with the following primers using EF-Taq polymerase (SolGent, Seoul, Korea): GM3 synthase (413 bp), 5′-CCCTGCCATTCTGGGTACGAC-3′ (sense) and 5′-CACGATCAATGCCTCCACTGAGATC-3′ (antisense); GD3 synthase (460 bp), 5′-TGTGGTCCAGAAAGACATTTGTGGA-3′ (sense) and 5′-TGGAGTGAGGTATCTTCACATGGGT-3′(antisense) GalNAc-T (GM2/GD2 synthase) (230 bp), 5′-CCAACTCAACAGGCAACTAC-3′(sense) and 5′-GATCATAACGGAGGAAGGTC-3′ (antisense); β-actin (247 bp), 5′-CAAGAGATGGCCACGGCTGCT-3′ (sense) and 5′-TCCTTCTGCATCCTGTCGGCA-3′ (antisense). The use of equal amounts of mRNA in the RT-PCR assay was confirmed by analyzing the expression levels of β-actin. The PCR products were separated by gel electrophoresis on 1.5% agarose-containing ethidium bromide with 0.5× Tris-acetate-EDTA (TAE) buffer.

### Cell viability assay

Cell proliferation was investigated using a commercially available proliferation kit II (XTT; Boehringer Mannheim, Mannheim, Germany). Briefly, the cells were sub-cultured in 96-well culture plates at a density of 103 cells/well in 100 µL of DMEM/10% FBS. After 24 h of incubation, the medium was discarded and replaced with 100 µL of fresh medium containing various concentrations of CDDP. The plates were incubated at 37°C in a humidified incubator containing 5% CO2 for 12 h. At end of the incubation, 50 µL of the XTT test solution was prepared by mixing 5 mL of XTT-labeling reagent and 100 µL of electron-coupling reagent, which was added to each well. After 4 h of incubation at 37°C and under 5% CO2 conditions, the absorbance was measured using an ELISA reader (Molecular Devices, Sunnyvale, CA) at a test wavelength of 490 nm.

### Flow cytometric estimation of intracellular redox state

ROS production was evaluated by staining cells with dichlorodihydrofluorescein diacetate (H_2_DCFDA; Molecular Probes, Carlsbad, CA). Cells were washed twice with DMEM containing 10% FBS, incubated in 10 µM H_2_DCFDA diluted in DMEM for 20 min at 37°C, washed with PBS, and trypsinized. Dissociated cells were washed twice with ice-cold PBS, resuspended in PBS, and analyzed by flow cytometry using FACS Calibur (Becton–Dickinson, Mountain View, CA).

### Western blot analysis

Cells were homogenized in a buffer containing 50 mM Tris-HCl (pH 8.0), 150 mM NaCl, 0.02% NaN_3_, 100 µg/mL phenylmethylsulfonyl fluoride (PMSF), 1 µg/mL aprotinin, and 1% Triton X-100. Protein concentrations were measured using the Bio-Rad protein assay (Bio-Rad, Richmond, CA). Thirty-micrograms of total cell lysate was size fractionated by sodium dodecyl sulfate-polyacrylamide gel electrophoresis (SDS-PAGE) and transferred onto nitrocellulose membranes using the Hoefer electrotransfer system (Amersham Biosciences, Buckinghamshire, UK). To detect the target proteins, we incubated the membranes with the respective antibodies. Detection was performed using secondary horseradish peroxidase-linked anti-mouse and anti-rabbit antibodies (Santa Cruz), and ECL chemiluminescence system (Amersham Biosciences).

### Over-expression of ganglioside GM3 synthase and its product in HCT116 cells

To construct the GM3 synthase expression plasmid, a 1.1 kb DNA fragment including the human GM3 synthase coding region was amplified by PCR using primer oligonucleotides 5′-CTAAGCTTATGAGAAGGCCCAGCTTGTTATTAAAAGACATC-3′ (sense), 5′-ATGAATTCGTTCAAAATTCACGATCAATGCCTCCACTGAGATC-3′ (antisense) and human fetal brain cDNA as a template. The sense and antisense primers contain *Hind* III and *Eco* RI restriction sites (underlined), respectively. The fragment was purified from a 1% agarose gel using the Wizard SV Gel and PCR Clean-Up System (Promega) and digested with the appropriate restriction enzyme, and ligated using T4 ligase (Takara Bio Inc., Shiga, Japan) into a pcDNA3 vector, to generate pcDNA-GM3. To identify the construct with GM3 synthase gene, restriction mapping and DNA sequencing were carried out. HCT116 cells were plated onto 6-well plates at density of 10^5^ cells/well and grown overnight. Cells were transfected with 1 µg of pcDNA and pcDNA-GM3 plasmid by WelFect-EX™ PLUS method (JBI). After incubation, the transfected cells were cultured in the presence of 500 µg/mL G418 (Life Technologies, Inc.). After 21 days in the selective medium, individual G418-resistant colonies were isolated. Three positive clones expressing GM3 synthase to high levels, as determined by RT-PCR, were used for further analysis.

### Luciferase assay

Reporter plasmids, pGL3-1600 were prepared by insertion of the *Sac* I/*Bgl* II fragments from the each of the plasmids generated previously [23] into the corresponding sites of the promoter-less luciferase vector pGL3-Basic (Promega). Cells were plated onto 6-well plates at density of 10^5^ cells/well and grown overnight. Cells were co-transfected with 0.5 pmol of GM3 synthase promoter-luciferase reporter constructs and 0.5 µg of β-galactosidase plasmid by WelFect-EX™ PLUS method (JBI). Cells were cultured in medium containing 10% FBS and incubated with CDDP for 12 h. Luciferase activity and β-galactosidase activity were assayed by using the luciferase and β-galactosidase enzyme assay system (Promega). Luciferase activity was normalized to the β-galactosidase activity in the cell lysate and the average was calculated based on three independent experiments.

### Immunofluorescence microscopy

HCT116 colon cancer cells were seeded at a sub-confluent density on 12 mm- diameter sterile coverslips in six-well tissue culture plates. Cells were fixed in 3.7% formaldehyde/PBS and washed three times with PBS and then permeabilized in 0.5% Tween-20/PBS for 5 min at room temperature. Non-specific sites were then blocked with PBS containing 1% bovine serum albumin for 30 min at room temperature with gentle rocking. Thereafter, a solution of GM3 (M2590), GD3 or GM2-specific antibodies were flooded over the cells at 4°C overnight. After washing with PBS, the cells were further incubated with FITC conjugated anti-mouse IgM for 30 min at room temperature, followed by washing with PBS, and finally mounted in anti-fade reagent (Molecular Probes) containing 4′,6-iamidino-2-phenylindole (DAPI). The slides were analyzed using a Nikon fluorescence microscopy (Nikon, Japan). The pre-absorbed secondary antibody alone was also included as a negative control for the experiment.

### DNA fragmentation assay

The cultured cells were harvested, washed with PBS, lysed in buffer (10 mM Tris-HCl (pH 7.4), 10 mM EDTA, 0.5% Triton X-100), and incubated on ice for 10 min. Samples were centrifuged at 4°C for 10 min, and supernatants were incubated with 200 µg/mL RNase A at 37°C for 1 h. Subsequently, the samples were incubated with 200 µg/mL protease K at 50°C for 30 min. After precipitation with isopropanol, DNA was resuspended in a Tris-EDTA solution. Samples were resolved by electrophoresis on a 2% agarose gel and visualized by ethidium bromide staining and UV transillumination.

### Flow cytometric analysis of apoptotic cells

To identify apoptotic cells, pcDNA- or pcDNA-GM3-transfectaed HCT116 cells were treated with CDDP for 24 h. The samples were washed with PBS, stained with 1 µg/mL of FITC-Annexin V (BD Pharmingen, San Diego, CA) for 10 min in the dark at room temperature and analyzed by flow cytometry using a FACS Calibur instrument (Becton-Dickinson).

### HPTLC analysis of gangliosides

Gangliosides from human colon cancer cells treated with or without CDDP (1.5×10^7^ cells) were extracted with chloroform: methanol: water (2∶1∶0.4, v/v/v), and were fractionated and analyzed by HPTLC using Silica Gel 60 plates (Merck). The plates were developed with chloroform: methanol: 0.2% aqueous CaCl_2_ (55∶45∶10, v/v/v). Gangliosides were visualized by spraying the plate with the resorcinol hydrochoride reagent.

### Preparation and transfection of small interference RNAs (siRNAs)

An siRNA duplex designed to target the coding sequence of human GM3 synthase mRNA and plant chlorophyll a/b-binding protein mRNA as a negative control were synthesized by Bioneer Corporation. The target sequences for GM3 synthase siRNAs are 5′-GUAUGUAACGAUGUUGUAU-3′, 5′-CAUCAAAGAGACUGCCUUU-3′, and 5′-CAGGUAUAGCGUGGACUUA-3′. HCT116 colon cancer cells were transfected with GM3 synthase siRNAs and negative control siRNA respectively, by using WelFect-EX™ PLUS (JBI), according to the manufacturer's instructions. One day after transfection, transfection complexes were removed and replaced with culture medium. After incubating with CDDP in culture medium for 24 h, the transfected cells were used for further analysis.

## Results

### CDDP induces apoptosis through the accumulation of ROS

**Figure 1:**
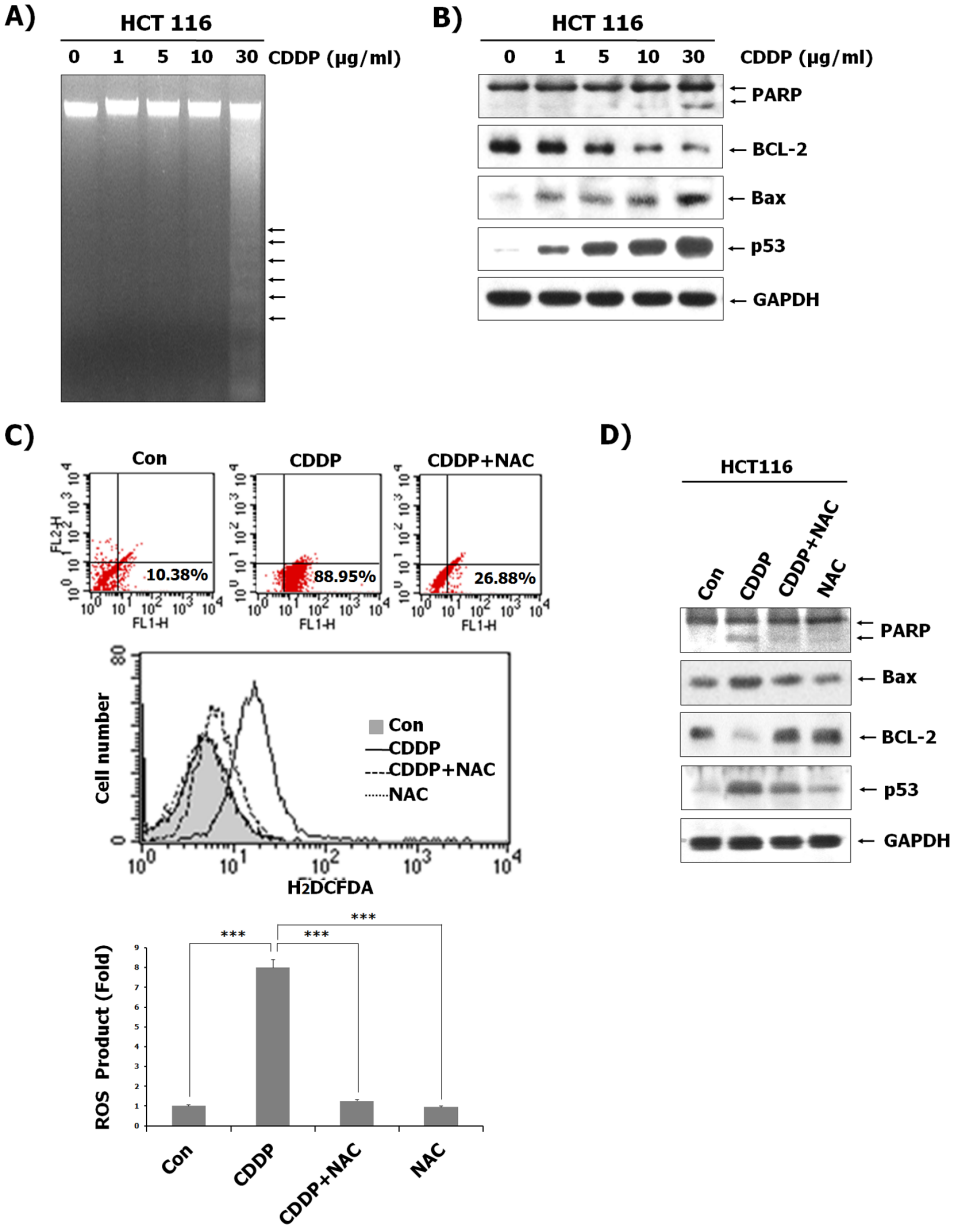
HCT116 cell apoptosis induced by CDDP via a ROS-dependent pathway. (A) After treatment of CDDP at the indicated concentrations for 24 h, genomic DNA was isolated and separated by electrophoresis on a 2% agarose gel. The DNA was stained with ethidium bromide and visualized under UV light. (B) Total protein from HCT116 cells was isolated, and immunoblotting analysis was performed with the indicated antibodies. (C) HCT116 cells were treated with or without 30 µg/mL CDDP after pre-treatment with 10 nM NAC, followed by the replacement of the culture medium with freshly prepared medium containing 10 µM DCFH-DA. After 30 min of incubation at 37°C, fluorescence intensity was measured by flow cytometry. Results represent the fold increase from their respective controls (cells not treated with CDDP), considered as 1. Data represent the mean ± SD of 3 independent measurements. ^***^P<0.001 vs. the CDDP-treated control. (D) HCT116 cells were treated with or without CDDP (30 µg/mL) after pre-treatment of NAC (10 nM). Protein was isolated from the cells and analyzed by immunoblotting using indicated antibodies. GAPDH was included as an internal control.

To determine the ability of CDDP to induce apoptosis in HCT116 cells, cells were treated with various concentrations of CDDP for 24 h and DNA isolated from CDDP-treated HCT116 cells were resolved by electrophoresis. As shown in Fig. 1, CDDP induced DNA fragmentation. We also examined the effect of CDDP on mitochondria-dependent apoptosis signals and the expression of p53 during CDDP-induced apoptosis. Treatment of HCT116 cells with CDDP resulted in the increase of p53 and Bax expression as well as the reduction of Bcl-2 expression. Furthermore, CDDP treatment resulted in increased cleavage of PARP, indicating caspase activation by the release of cytochrome c through the reduction of mitochondrial membrane potential (Fig. 1B). As the generation of ROS has been reported to play an important role in CDDP-induced cytotoxicity [24], the effect of CDDP treatment on intracellular redox status of HCT116 cells was assessed. We compared the production of ROS in the CDDP-treated cells to that in CDDP-untreated cells by flow cytometric analysis using H_2_DCFDA. As shown in Fig. 1C, ROS production was increased in CDDP- treated cells compared to CDDP- untreated cells. However, concomitant treatment with NAC, an ROS inhibitor, along with CDDP reduced the accumulation of intracellular ROS (Fig. 1C). Correlated with the inhibitory ability to induce ROS, NAC was also able to significantly inhibit cell death signals in the presence of CDDP, as evidenced by Western blotting. As shown in Fig. 1D, CDDP induced the expression of the apoptotic proteins Bax and p53 as well as induced PARP cleavage and the reduced expression of anti-apoptotic Bcl-2 protein. These effects of CDDP on human colon cancer cells were blocked by NAC. These results indicate that generation of ROS by CDDP is required for apoptosis of HCT116 cells.

### CDDP increases ganglioside GM3 in colon cancer cells

**Figure 2:**
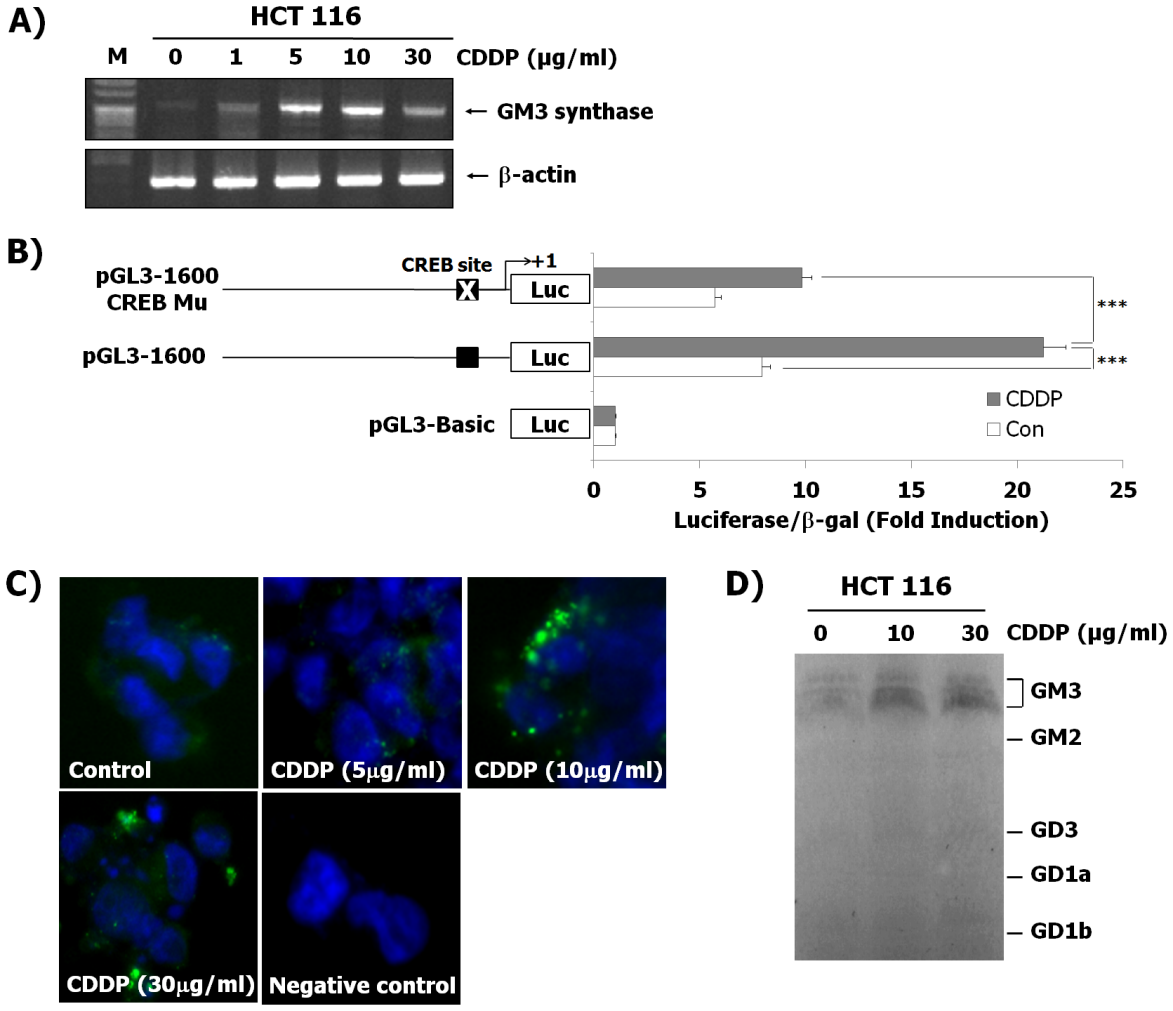
Enhanced expression of GM3 synthase and its product GM3 in HCT116 cells after CDDP treatment. (A) Total RNA from HCT116 cells was isolated following treatment with the indicated concentrations of CDDP treatment for 12 h and GM3 synthase mRNA was detected by RT-PCR. (B) HCT116 cells were transiently transfected with GM3 synthase gene promoter (pGL3-1600) and CREB mutation of GM3 synthase promoter (pGL3-1600 CREB Mu), and then cultured with or without CDDP for 12 h. Luciferase activity was determined from cell lysates as described in the Materials and Methods. The results shown are means ± SD of three independent experiments with triplicate measurement. ^***^P<0.001 vs. the CDDP-treated control. (C) HCT116 cells were cultured and then incubated with indicated concentration of CDDP treatment for 12 h. Immunoreactivity against ganglioside GM3 was detected using a fluorescein isothiocyanate (FITC)-conjugated goat anti-mouse IgM without or with the M2590 antibody. (D) The gangliosides isolated from the HCT116 cells treated with CDDP (0, 10, and 30 µg/mL) were analyzed by HPTLC.

Previous studies have reported that cell apoptosis is caused by the alteration of membrane composition [13], [14].We examined whether CDDP in HCT116 cells regulates expression levels of GM2 and GD3 synthase genes and the gangliosides of GM2 and GD3, but there were no changes both in the parameters of gene expression (data not shown) and ganglioside production (Fig. 2D). CDDP increased the expression of GM3 synthase only in the CDDP-induced HCT116 cells (Fig. 2A). In addition to this, we also investigated whether CDDP induces the expression of GM3 synthase using other colon cancer cells such as DLD-1, LoVo, and WiDr. CDDP treatment resulted in the enhanced expression of GM3 synthase in other human colon cancer cell lines (data not shown). We further investigated whether expression of GM3 synthase in the present model of human colon cancer HCT116 cells is induced by the use of other chemotherapeutic agents (5-Fluorouracil and Doxorubicin) which are well- known to induce apoptosis of various human cancer cells as well as colon cancer cells. The expression of GM3 synthase was not induced by both anti-cancer agents (data not shown). As shown in Fig. 2A, mRNA levels of GM3 synthase were increased in HCT116 cells induced by CDDP, as evidenced by RT-PCR. In addition, we investigated whether the promoter activity of GM3 synthase gene is stimulated in CDDP-induced HCT116 cells. Recently, we have reported the CREB-mediated transcriptional regulation of the human GM3 synthase gene [23]. Thus, to determine GM3 synthase gene promoter activity by CDDP, GM3 synthase gene promoter (pGL3-1600), CREB mutation of GM3 synthase promoter (pGL3-1600 CREB Mu) and pGL3-basic plasmids were transfected into HCT116 cells with, or without CDDP, respectively, followed by measurement of luciferase activity using a reporter assay. As shown in Fig. 1B, the transcriptional activity of pGL3-1600 in HCT116 cells treated with CDDP was significantly higher than that in the untreated control cells and in the pGL3-Basic-transfected HCT116 cells with or without CDDP treatment. As expected, transcriptional activity from the CREB mutant markedly decreased by about two-fold compared to pGL3-1600 promoter in CDDP-treated HCT116 cells. Furthermore, as shown in Fig. 2C, an increase in ganglioside GM3 production was observed in HCT1116 cells induced by CDDP, as evidenced by immunofluorescence assay. Immunofluorescence activity of GM3 in CDDP-treated cells became highly detectable in a dose-dependent manner, while that in the untreated cells was not detected, using M2590 as a GM3-specific monoclonal antibody and secondary antibody as a negative control, respectively. Moreover, we investigated ganglioside patterns in the HCT116 cells treated with and without CDDP using HPTLC analysis. Interestingly, among the gangliosides, ganglioside GM3 was markedly increased in HCT116 cells treated with CDDP compared the no CDDP control (Fig. 2D). These results clearly show that both the GM3 synthase expression and ganglioside GM3 production are increased in HCT116 cells treated with CDDP.

### Down-regulation of ganglioside GM3 by siRNA targeting GM3 synthase protects cell apoptosis by CDDP

**Figure 3:**
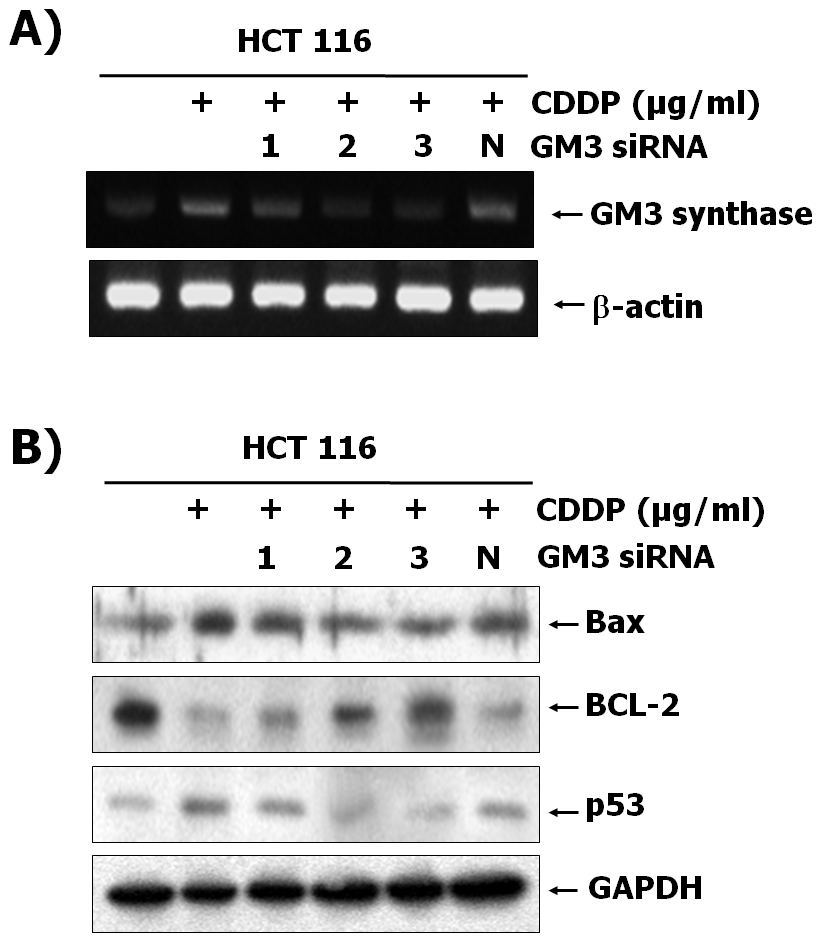
Suppression of apoptotic signal events by GM3 RNA interference in CDDP-exposed cells. HCT116 cells were transfected with three siRNA targeting GM3 and negative control siRNA. Cells were then cultured for 12(30 µg/mL). Total RNA and protein lysates from the cells were prepared as described in Materials and Methods. (A) The levels of GM3 synthase mRNA in total RNA obtained from each cell was detected by RT-PCR. (B) Total protein was prepared from the cells and analyzed by western blotting using the indicated antibodies.

Our previous data showed that CDDP induced apoptotic signal events through ROS production, and enhanced expression of ganglioside GM3 synthase and its product GM3. Thus, to determine whether the increased ganglioside GM3 in CDDP-treated HCT116 cells was associated with CDDP-mediated apoptosis, we designed three siRNAs directed specifically against GM3 synthase (siGM3). No. 2 and 3 but not No. 1 of three siGM3 clearly down-regulated expression of GM3 synthase in CDDP-treated HCT116 cells, as determined by RT-PCR analysis (Fig. 3A). In addition, siGM3 2 and 3 but not 1 (Fig. 3B) restored expression patterns of Bax, Bcl-2 and p53 in CDDP-treated cells to those in CDDP-uninduced cells. These results suggest that the up-regulation of GM3 is required for apoptosis of CDDP-induced HCT116 cells.

### The enhancement of ganglioside GM3 in CDDP-treated HCT116 cells regulates 12-lipoxigenase (12-LOX) activation for ROS production

**Figure 4:**
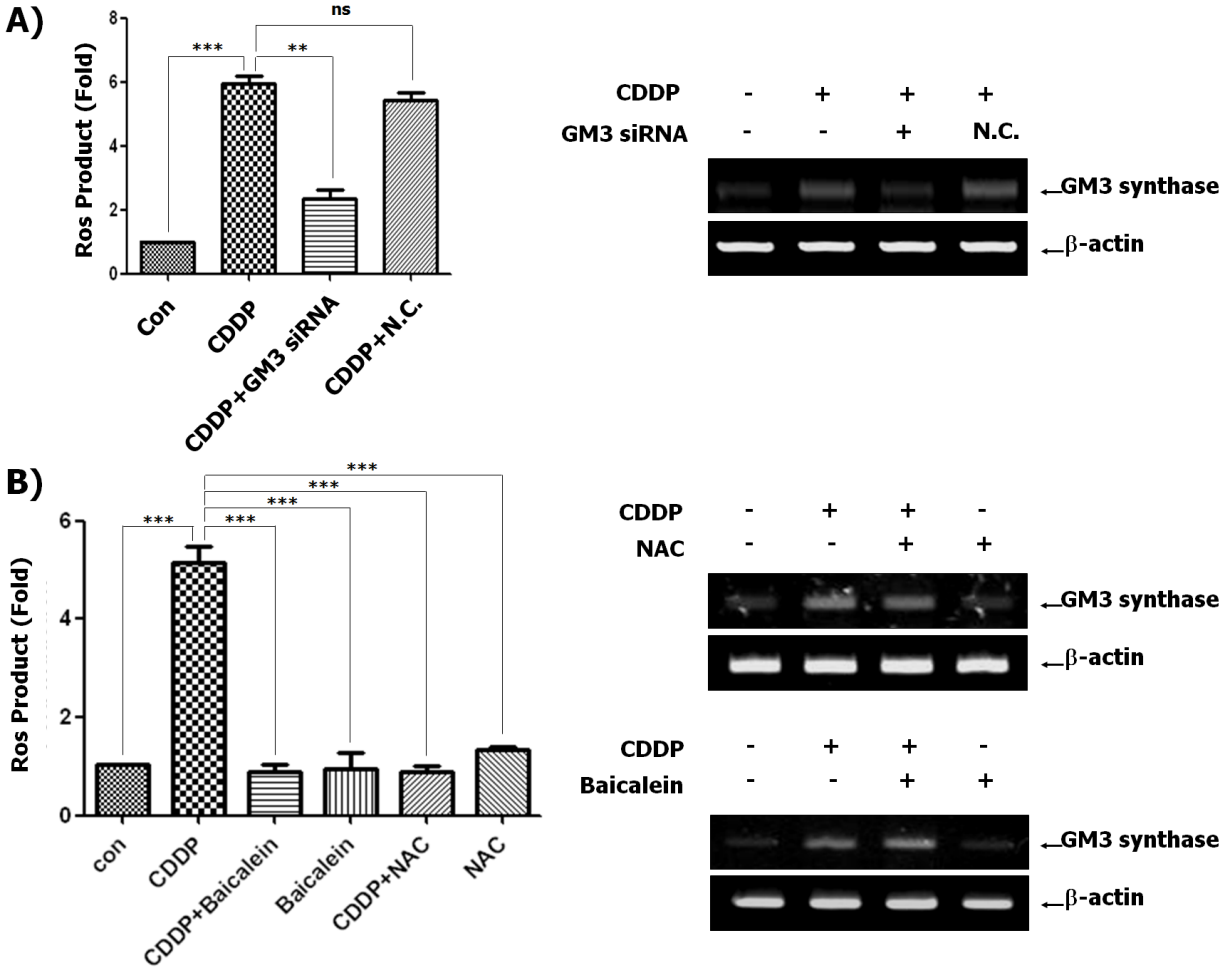
Regulation of 12-LOX activity by CDDP-induced ganglioside GM3 in HCT116 cells. (A) HCT116 cells were transfected with siRNA targeting GM3 and negative control siRNA. The cells were then cultured for 12 h in the presence or absence of CDDP (30 µg/mL). The culture medium was replaced with freshly prepared medium containing 10 µM DCFH-DA. After 30 min of incubation at 37°C, fluorescence intensity was measured by flow cytometry. Data represent the mean ± SD of 3 independent measurements. ^**^P<0.01 and ^***^P<0.001 vs. the CDDP-treated control. ns: no significance. For RT-PCR, total RNA and protein lysates from the cells were prepared as described in Materials and Methods. The levels of GM3 synthase mRNA in total RNA obtained from each cell were detected by RT-PCR. (B) HCT116 cells were treated with or without 30 µg/mL CDDP after pre-treatment of 10 nM NAC or 1 µM Baicalein, and then culture medium was replaced with freshly prepared medium containing 10 µM DCFH-DA. After 30 min of incubation at 37°C, fluorescence intensity was measured by flow cytometry. Data represent the mean ± SD of 3 independent measurements. ^***^P<0.001 vs. the CDDP-treated control. For RT-PCR, total RNA and protein lysates from the cells were prepared as described in Materials and Methods. The levels of GM3 synthase mRNA in total RNA obtained from each cell were detected by RT-PCR.

Nerve cell death induced by glutamate results in ROS production through 12-LOX activation, and induced localization of 12-LOX to the membrane, which is related to activation of 12-LOX [25]. Our colleagues have reported that 12-LOX is recruited to glycosphingolipid-enriched microdomains (GEM) in a ganglioside GM3-dependent manner during oxidative glutamate toxicity [21]. Our data show that CDDP induced mitochondria-dependent apoptosis signals through ganglioside GM3-mediated ROS generation in HCT116 cells (Fig. 3 and Fig. 4A). Thus, we investigated whether a specific inhibitor of 12-LOX, Baicalein inhibits 12-LOX activity for ROS production in HCT116 cells expressing ganglioside GM3 induced by CDDP. As shown in Fig. 4B, CDDP induced GM3 synthase expression and ROS production. However, Baicalein suppressed CDDP-stimulated ROS production, although GM3 synthase expression induced by CDDP was not regulated by Baicalein. Furthermore, the ROS scavenger NAC, inhibited ROS generation, but not GM3 synthase in CDDP-treated HCT116 cells. These results suggest that CDDP-induced ganglioside GM3 expression may regulate 12-LOX activity for ROS production through 12-LOX recruitment to GEM.

### CDDP-induced apoptosis is enhanced in HCT116 cells that overexpress GM3 synthase

**Figure 5:**
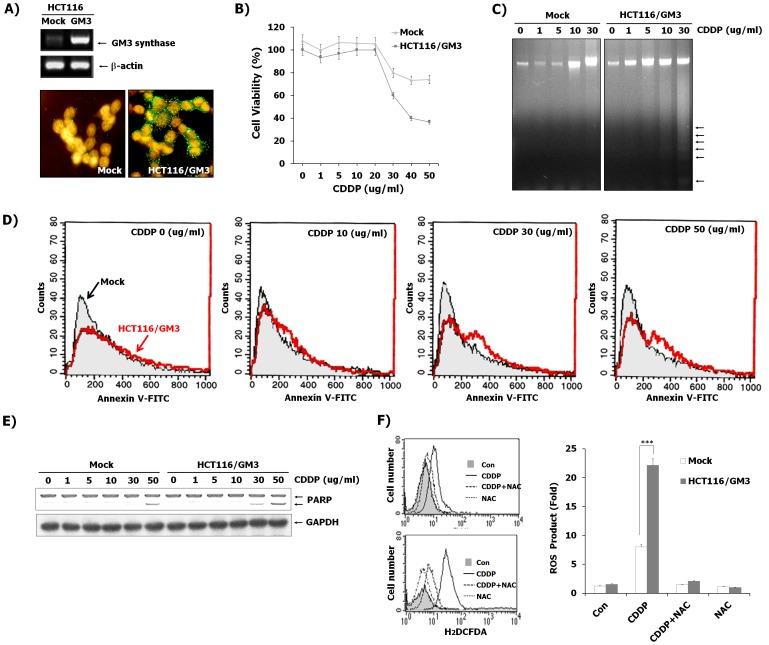
The effect of overexpression of GM3 in HCT116 cells on CDDP-induced cytotoxicity. (A) RT-PCR and immunofluorescence assay confirmed the expression of GM3 synthase and its GM3 product in a stable transfectant of pcDNA-GM3 in HCT116 cells. As a control, vector alone (pcDNA)-transfected HCT116 did not express GM3. Mock, vector (pcDNA3)-transfected cell HCT116/GM3, pcDNA3-GM3-transfected cells. (B) Cell viability was evaluated 24 h after CDDP treatment at the indicated concentrations using XTT assay in HCT116/GM3 cells compared to mock-treated cells. Data are means ± SD of three independent experiments. (C) After treatment of CDDP at the indicated concentrations for 24 h, genomic DNA was analyzed on a 2% agarose gel. (D) Apoptotic cell populations were measured by FACS Calibur analysis employing Annexin V-staining as described in the Materials and Methods. Arrows indicate apoptotic populations in mock and HCT116/GM3 cells, respectively. (E) HCT116/GM3 or mock cells were treated with indicated the concentrations of CDDP for 12 h. Whole-cell lysates and supernatant media were prepared and analyzed by immunoblotting as described in Materials and Methods, using the indicated antibodies. (F) The effect of CDDP treatment was measured on the fluorescence distribution of DCFH oxidation in HCT116/GM3 or mock cells. Cells from each condition were cultured after pretreatment with NAC (10 nM) for 12 h in the absence or presence of CDDP (30 µg/mL), following which culture medium was replaced with freshly prepared medium containing 10 µM DCFH-DA. After 30 min of incubation at 37°C, fluorescence intensity was measured by flow cytometry. Values are apoptotic intensity as folds of control and present means ± SD. ^***^P<0.001 vs. the CDDP-treated control in Mock-transfected cells.

To examine the sensitivity of CDDP-mediated apoptosis between HCT116 cells and HCT116 cells overexpressing GM3 synthase, we generated stable transfectants expressing GM3. The pcDNA3-GM3 expression vector harboring the human GM3 synthase cDNA (HCT116/GM3) and pcDNA3 empty vector (mock) were transfected into HCT116 cells. After selection of G418-resistant colonies, we confirmed that the expression of GM3 synthase mRNA and ganglioside GM3 were highly expressed in HCT116/GM3 cells compared to mock-transfected cells, as evidenced by RT-PCR and immunofluorescence assay (Fig. 5A). These results show that the enzymatically active GM3 synthase is synthesized in HCT116/GM3 cells. Under normal culture conditions (10% FBS), the growth rate of HCT116/GM3 cells was not different compared to control cells, and changes in apoptotic signals were not observed in these cells, as evidenced by western blot analysis (data not shown). However, when these transfectants were treated with CDDP in a dose-dependent manner, higher numbers of the cells dissociated from the plate in HCT116/GM3 compared to mock cells treated with 30 µg/mL CDDP. Indeed, HCT116/GM3 cells were shown to be more sensitive to CDDP than mock cells in cell growth inhibition as evidenced by XTT assay (Fig. 5B). In addition, an increase in DNA damage after CDDP exposure was observed in HCT116/GM3 cells compared to mock-treated cells using a DNA fragmentation assay (Fig. 5C). Since Annexin V stains the flipped out phosphatidylserine on the outer side of the plasma membrane, flipping out of the phosphatidylserine from the inner side to the outer side of the plasma membrane is known to be one of the characteristics of apoptotic cells. In the present study, as shown in Fig. 5D, annexin V positive cells measured by flow cytometry were also significantly increased in CDDP-treated HCT116/GM3 cells compared with mock cells. To investigate the molecular mechanism underlying the sensitizing effect of GM3 in CDDP-induced cytotoxicity, the caspase-mediated PARP degradation by reduction of mitochondrial potential was examined after CDDP treatment at various concentrations (Fig. 5E). Western blot analysis showed that in HCT116/GM3 cells the cleavage level of PARP was increased compared to mock cells. These results suggest that GM3 sensitizes HCT116 cells to CDDP-induced apoptosis. Our results showed that ROS plays a central role in CDDP-induced HCT116 cells, leading to cell apoptosis. We compared the production of ROS in HCT116/GM3 cells to that in mock cells by flow cytometric analysis using H_2_DCFDA. As shown in Fig. 5F, the CDDP-induced ROS production was augmented in HCT116/GM3 cells compared to mock cells, but not in NAC-pretreated cells, indicating that an increase in GM3 levels is required for the ROS production in CDDP-induced apoptosis of HCT116 cells. These data indicate that ROS production is dependent on GM3 levels in CDDP-induced HCT116 cells.

## Discussion

CDDP is widely used for the treatment of many malignancies [26]–[29]. The cytotoxic mechanism of CDDP, a well-known DNA-damaging agent, is through the induction of DNA adducts, protein-protein interactions, increased membrane fluidity, and a subsequent induction of apoptosis [30]–[32]. However, the mediator involved in the induction of apoptosis following exposure to CDDP is still poorly understood. In this study, our results indicate that CDDP significantly enhances GM3 expression, which results in apoptosis induction in HCT116 cells, thus clarifying a crucial mechanism(s) involving the oxidative stress-mediated mitochondrial pathway. Additionally, the overexpression of GM3 sensitizes HCT116 cells to CDDP-induced apoptosis through the cellular accumulation of ROS.

Apoptosis can generally occur through the death receptor and the mitochondrial pathways [33]. In the cell surface death receptor pathway, when the death receptors bind to specific ligands to form the death-inducing signaling complex, intracellular signals are activated to eventually lead to cell death. In the mitochondrial pathway, changes in Bcl-2 family member levels modulate the mitochondrial membrane permeability and induce release of cytochrome *c* and apoptosis-inducing factors from the mitochondria, leading to cleavage of PARP and ultimately DNA fragmentation. Our data show that CDDP leads to apoptosis in human colon cancer cells and promotes apoptosis of HCT116 cells through mitochondrial signaling. For evidence of apoptosis, Western blot analysis revealed an increase in PARP cleavage and the Bax levels with a decrease in Bcl-2 levels. This is characteristic of the mitochondria-dependent cell death pathway (Fig. 1). Additionally, DNA damage caused by the activation of caspases through a change in mitochondrial membrane permeability resulted in cell apoptosis, as evidenced by the DNA fragmentation assay (Fig. 1). The generation of ROS leads to multiple apoptotic signals, including caspase-dependent and –independent signals [12]. We also observed that CDDP induces cellular accumulation of ROS in HCT116 cells, as determined by flow cytometric analysis using H_2_DCFDA, and the concomitant administration of NAC, a ROS inhibitor, with CDDP diminished the accumulation of intracellular ROS (Fig. 1). Moreover, NAC inhibited the CDDP-induced expression of the apoptotic proteins Bax and p53, as well as PARP cleavage, and reduced the CDDP-induced expression of the anti-apoptotic Bcl-2 protein in human colon cancer cells (Fig. 1). These results indicate that generation of ROS by CDDP is required for the apoptosis of HCT116 cells.

GSLs are membrane lipids and are ubiquitously present in the lipid raft, caveolae, or GSL-enriched microdomains (GEM) in the plasma membranes of all mammalian cells. Because of clustering of GSLs with several receptors or signal molecules, the changes in GSLs composition play significant roles in cell adhesion/recognition processes and in initiation/modulation of signal transduction [15], [34]–[36]. It is well known that gangliosides, which are sialic acid-containing glycosphingolipids, have been implicated in the regulation of cell proliferation and cell death. Our group has also reported that GM3 has an effect on cell cycle arrest through up-regulation of PTEN in HCT116 cells, while GM1 and lactosylceramide do not [37]. In addition, the ganglioside GM3 induces apoptosis of high-grade human glioblastoma multiform tumors [19]. In hepatocytes and hematopoietic cells, GD3 also causes apoptosis of cells, but GM3 and GT1b do not [38], [39]. Disialosyl-gangliosides GD3 and GD1b induce apoptosis in human colon cancer Colo-205 and breast carcinoma SKBR3 cells [40]. Furthermore, up-regulation of plasma membrane-associated ganglioside sialidase (Neu3) in human colon cancer leads to protection against apoptosis through a marked accumulation of lactosylceramide, a possible sialidase product instead of gangliosides and ceramide associated with apoptosis of cancer cells [41]. These reports suggest that gangliosides induce cell apoptosis in a manner that is cell-specific and ganglioside-specific. On the other hand, treatment with the glucosylceramide synthesis inhibitors PDMP or PPMP results in the apoptosis of Colo-205 and SKBR3 cells through the accumulation of ceramide, which is a known apoptosis inducer in cancer cells, although the synthesis of the apoptosis-inducing gangliosides is inhibited by PDMP or PPMP [42]. In CDDP-resistant lung cancer SBC-3 cells, ganglioside GM3 is markedly increased [43]. However, Nojiri and colleagues showed that Brefeldin A, a known anti-cancer drug, clearly increases the ganglioside GM3 proportion among glycolipids in HCT116 cells, resulting in the inhibition of tumor growth [44]. We also found CDDP remarkably increases transcription level of GM3 synthase and GM3 product in HCT116 cells, suggesting that the endogenously elevated GM3 causes cell death (Fig. 2). In our previous study, we demonstrated that transcriptional activation of GM3 synthase in phorbol 12-myristate 13-acetate (PMA)-stimulated HL-60 cells was associated with protein kinase C-dependent CREB activation [45]. Rubin et al. have reported that the induction of DNA-damaged apoptosis by CDDP involved the PKC-dependent signaling pathway [46]. Our data showed that the transcriptional activity of pGL3-1600 in CDDP-treated HCT116 cells was significantly increased compared to CDDP-untreated cells. However, transcriptional activity from CREB mutant in CDDP-treated HCT116 cells was markedly decreased compared to pGL3-1600 promoter in CDDP-treated HCT116 cells (Fig. 2B). Thus, to elucidate how CDDP might increase GM3 synthase expression in colon cancer HCT116 cells, we continuously find precise signal pathways and transcriptional factors related to expression of GM3 synthase induced by CDDP. Furthermore, to clarify whether GM3 plays an important role in CDDP-induced apoptosis of HCT116, siRNA-mediated silencing of GM3 synthase was applied for down-regulated synthesis of GM3 in the cells. GM3 synthase siRNAs significantly restored increased expression of Bax, reduction of Bcl-2 expression, and PARP cleavage related to apoptotic signals in CDDP-treated HCT116 cells to those in CDDP-uninduced cells (Fig. 3).

It is well-known that CDDP induces ROS production in mitochondria through cytoplasmic Nox enzymes [47]–[49]. Furthermore, Brightman et al. have already reported that ganglioside GM3 regulated activity of Nox enzyme [50]. Thus, to further evaluate the importance of these findings by linking a mechanism to the changes in ROS levels by CDDP, we have investigated the mechanism. It is known that ROS are generated by arachidonic acid (AA) metabolites of phospholipase A2 and LOX enzymes [51]. In drug-induced cell toxicity, 12-LOX activation and ROS accumulation are increased [21], [52], [53]. For cellular localization of 12-LOX, increased 12-LOX activity is associated with the translocation of soluble 12-LOX to membranes [21], [53], [54]. Moreover, our collaborating results show that 12-LOX is recruited to glycosphingolipid-enriched microdomains (GEM) in a ganglioside GM3-dependent manner during oxidative glutamate toxicity, resulting in overproduction of 12-LOX metabolite, 12-HETE, associated with ROS production in glutamate-induced cell death [21]. Our present data clearly show that CDDP induces mitochondria-dependent apoptosis signals by regulating Bcl-2 and Bax expression through ganglioside GM3-mediated ROS generation in HCT116 cells (Fig. 1B, Fig. 3 and Fig. 4A). Thus, we investigated whether Baicalein, known as specific inhibitor of 12-LOX inhibits 12-LOX activity for ROS production in HCT116 cells when ganglioside GM3 is upregulated by CDDP. As shown in Fig. 4B, CDDP induced both GM3 synthase expression and ROS production. However, Baicalein suppressed CDDP-stimulated ROS production, although GM3 synthase expression induced by CDDP was not affected by Baicalein. Furthermore, the ROS scavenger NAC inhibited ROS generation, but not GM3 synthase expression in the CDDP-treated HCT116 cells. These results suggest that CDDP-induced production of ganglioside GM3 may regulate 12-LOX activity for ROS production through 12-LOX recruitment to GEM.

It has been reported that ROS generation leads to the induction of the pro-apoptotic protein p53 [12] and also suppresses the expression of Bcl-2, but increases expression of Bax [55]. Furthermore, tumor suppressor p53 is a regulator of Bcl-2 and Bax gene expression in vitro and in vivo, resulting in decrease in expression of the apoptosis-suppressing gene Bcl-2 and increase in the expression of Bax, a gene which encodes a dominant inhibitor of the Bcl-2 protein [56]. Our present data shows that CDDP regulates p53, Bcl-2 and Bax expression resulting in mitochondrial damage through ganglioside GM3-mediated ROS generation in HCT116 cells (Fig. 1B, Fig. 3 and Fig. 4A).

In order to examine GM3 function in the cells, we overexpressed GM3 synthase in HCT116 cells. The apoptotic effect was highly detected in HCT116/GM3 cells after CDDP exposure compared to mock cells. In presence of CDDP, HCT116/GM3 cells induced cell apoptosis compared to mock-treated cells, as shown in cell proliferation, DNA fragmentation assay, Annexin V staining and PARP cleavage. Additionally, it was shown that apoptosis of HCT116/GM3 cells is associated with the ROS-mediated pathway. Our present results also showed the elevation of intracellular ROS in HCT116/GM3 cells exposed to relatively low concentration of CDDP. These data demonstrated the sensitizing effect of GM3 in HCT116 cells to CDDP is directly linked with accumulation of ROS.

In conclusion, we demonstrate that CDDP increases GM3 enrichment in cell membranes, and the increase of GM3 accelerates CDDP-induced cell killing. Consequently, GM3 expression may be related to the chemo-sensitivity of clinical samples of colon cancer, and our results suggest that its targeted expression may improve the efficiency of cancer therapies.
